# Endothelial activation and stress index (EASIX) as a predictor of acute kidney injury in patients undergoing transcatheter aortic valve replacement: A retrospective cohort study: Erratum

**DOI:** 10.1097/MD.0000000000050457

**Published:** 2026-10-09

**Authors:** Yasin Özen, Hüseyin Tezcan, Halil Özalp, Adnan Karaibrahimoglu, Kaan Burak Yildiz, Mevlüt Serdar Kuyumcu, Abdullah Tunçez, Kadri Murat Gürses, Mustafa Bilal Özbay, Ahmet Göktuğ Ertem, Muhammed Ulvi Yalçin, Nazif Aygul

In the article “Endothelial activation and stress index (EASIX) as a predictor of acute kidney injury in patients undergoing transcatheter aortic valve replacement: A retrospective cohort study,”^[1]^ which appears in Volume 105, Issue 10 of *Medicine*, table values were inadvertently published incorrectly, and some text changes have also been newly made. Overall, the following changes were made to the online version of the article.

1. The study cohort and CIN incidence reported in the Abstract and Results section have been corrected to reflect the final study cohort of 161 patients and 31 CIN events (19.3%).
2. The Statistical Analysis section has been corrected to accurately describe the final regression strategy and variable selection.
3. The previously reported intermediate regression model containing the GFR ratio has been removed. The final multivariable model was reconstructed using Contrast volume and EASIX, in accordance with the predefined Statistical Analysis plan. The corrected multivariable model showed that Contrast volume (OR = 1.148, 95% CI: 1.086–1.213, P <.001) and EASIX (OR = 2.490, 95% CI: 1.498–4.138, P <.001) remained significantly associated with CIN.
4. The AVA (aortic valve area) value in Table 1, which was inadvertently reported incorrectly, has been corrected.
5. The confidence interval, specificity, and sensitivity values reported in Table 2 have been corrected.
6. We revised the Results section and Abstract so that the reported odds ratios are consistent with the final regression model aligning with Table 3 values.
7. An expanded Study Limitations section has been added to the end of the Discussion, in accordance with the Editor’s request.
8. The CIN positive value in Figures 1 and 2 and their captions have changed from “>25%” to “≥25%.” Also, the AUC value in Figure 3 and its caption has changed from “0.910” to “0.908.” In this process, Figures 1 to 3 have been completely replaced with the updated figures.

**Table 1:** Demographic and clinical features of patients according to CIN status.

Parameter	CIN(−; n = 130)	CIN(+; n = 31)	*P*
Age (yr)	77.15 ± 8.17 (78.50; 72.75–83)	75.03 ± 9.07 (73; 70–81)	.206
Sex, male, n (%)	61 (46.9)	16 (51.6)	.640
Heart failure (HF), n (%)	30 (23.1)	10 (32.3)	.289
Hypertension (HT), n (%)	88 (67.7)	20 (64.5)	.832
Diabetes mellitus (DM), n (%)	60 (46.2)	21 (67.7)	.031*
Stroke, n (%)	4 (3.1)	3 (9.7)	.106
Atherosclerotic kidney disease (ASKVH), n (%)	37 (28.5)	3 (9.7)	.036*
ACEI/ARB usage, n (%)	90 (69.2)	16 (51.6)	.091
Thiazide diuretic usage, n (%)	26 (20.0)	7 (22.6)	.750
MRA usage, n (%)	15 (11.5)	5 (16.1)	.488
Left ventricular ejection fraction (LVEF, %)	49.11 ± 14.28 (55; 43.50–60)	47.94 ± 15.89 (55; 32–60)	.688
Aortic valve area (AVA, cm^2^)	0.70 +/-0.16 (0.70; 0.60-0.80)	0.71 +/-0.18 (0.70; 0.60-0.80)	.893
Maximum gradient (mm Hg)	75.40 ± 19.88 (72; 60–87.25)	79.35 ± 23.98 (77; 60–95)	.342
Mean gradient (mm Hg)	48.39 ± 13.55 (46; 40–56)	51.32 ± 16.51 (46; 40–66)	.365
WBC (10^3^/µL)	7.12 ± 2.03 (6.87; 5.85–8.29)	7.86 ± 2.67 (7.32; 5.53–9.30)	.092
Hemoglobin (Hb, g/dL)	11.89 ± 1.84 (12.05; 10.57–13.02)	11.98 ± 1.27 (11.80; 11.20–12.60)	.738
Hematocrit (Hct, %)	36.59 ± 5.34 (36.35; 32.87–39.92)	36.88 ± 3.68 (36.90; 34.80–38.20)	.722
Neutrophils (neu, 10^3^/µL)	4.76 ± 1.75 (4.30; 3.54–5.71)	5.60 ± 2.59 (4.69; 3.62–6.99)	.096
Lymphocytes (lymph, 10^3^/µL)	1.61 ± 0.63 (1.65; 1.09–2.04)	1.43 ± 0.70 (1.32; 0.89–1.73)	.151
Neutrophil-to-lymphocyte (N/L) ratio	3.47 ± 2.25 (2.75; 2.09–3.82)	5.00 ± 3.71 (4.03; 2.19–5.32)	.035**
Glucose (mg/dL)	121.91 ± 49.95 (108; 89.75–134)	150.71 ± 85.83 (121; 94–178)	.081
HbA1c (%)	6.42 ± 1.13 (6.20; 5.70–6.90)	6.93 ± 1.21 (6.50; 5.90–7.80)	.041**
Baseline GFR (mL/min/1.73 m^2^)	61.51 ± 19.02 (60; 50–78)	68.06 ± 25.95 (68; 48–91)	.194
48-h GFR (mL/min/1.73 m^2^)	66.86 ± 20.01 (68.50; 53–83)	48.43 ± 24.14 (45; 29–69)	<.001***
Baseline/48-h GFR ratio	1.10 ± 0.23 (1.05; 0.92–1.21)	0.69 ± 0.15 (0.68; 0.59–0.77)	<.001**
Baseline creatinine (mg/dL)	1.20 ± 0.72 (1.08; 0.87–1.26)	1.58 ± 0.70 (1.56; 1.14–1.76)	.010**
Platelets (PLT, 10^3^/µL)	235.95 ± 83.51 (219; 174–276.25)	185.39 ± 33.33 (183; 160–202)	<.001**
LDH (U/L)	229.01 ± 61.55 (217.50; 183–262.25)	345.00 ± 87.05 (362; 285–426)	<.001**
48-h creatinine (mg/dL)	1.06 ± 0.73 (0.93; 0.76–1.15)	4.22 ± 13.33 (1.32; 1.04–2.11)	.222
Baseline/48-h creatinine ratio	0.93 ± 0.16 (0.92; 0.80–1.07)	4.86 ± 17.62 (1.50; 1.35–1.65)	.249
Potassium (mEq/L)	4.42 ± 0.45 (4.50; 4.13–4.70)	4.48 ± 0.43 (4.40; 4.20–4.80)	.514
Triglycerides (mg/dL)	116.90 ± 48.06 (98.50; 81.75–151.25)	142.87 ± 89.65 (111; 82–180)	.128
HDL (mg/dL)	43.37 ± 13.43 (41; 33–52.22)	41.16 ± 11.95 (38; 32–51)	.402
LDL (mg/dL)	95.28 ± 37.28 (90.50; 66–115.50)	98.52 ± 30.94 (98; 74–116)	.656
BNP (pg/mL)	3366.53 ± 6838.51 (687; 196.75–2826)	3231.84 ± 4578.59 (1122; 641–3554)	.917
CRP (mg/L)	17.01 ± 28.45 (5; 3–20.25)	15.70 ± 22.35 (4; 3–14.70)	.812
Albumin (g/dL)	38.96 ± 4.15 (39.90; 36–42)	38.52 ± 5.00 (39; 34–43)	.647
CRP/albumin ratio	0.46 ± 0.77 (0.12; 0.07–0.54)	0.41 ± 0.61 (0.12; 0.06–0.35)	.747
CHADS-VASc score	3.81 ± 1.33 (4; 3–5)	3.58 ± 1.06 (4; 3–4)	.649
Contrast volume (mL)	135.62 ± 8.42 (135; 130–140)	150.97 ± 13.00 (150; 140–160)	<.001**
EASIX	1.33 ± 1.30 (1.07; 0.72–1.47)	2.92 ± 1.29 (2.60; 2.20–3.33)	<.001***

ACEI = angiotensin-converting enzyme inhibitor, ARB = angiotensin receptor blocker, ASKVH = atherosclerotic kidney disease, AVA = aortic valve area, BNP = B-type natriuretic peptide, CHADS-VASc = congestive heart failure, hypertension, age ≥75, diabetes mellitus, stroke, vascular disease, age 65–74, sex category, CIN = contrast-induced nephropathy, CRP = C-reactive protein, DM = diabetes mellitus, EASIX = Endothelial Activation and Stress Index, GFR = glomerular filtration rate, Hb = hemoglobin, Hct = hematocrit, HDL = high-density lipoprotein, HF = heart failure, HT = hypertension, LDH = lactate dehydrogenase, LDL = low-density lipoprotein, LVEF = left ventricular ejection fraction, Lymph = lymphocytes; N/L, neutrophil-to-lymphocyte, MRA = mineralocorticoid receptor antagonist, Neu = neutrophils, PLT = platelets, WBC = white blood cells.

* Significant at the.05 level according to chi-square test.

** Significant at the.05 level according to Student’s *t*-test.

*** Significant at the.05 level according to Mann–Whitney *U* test.

**Table 2:** ROC analysis of EASIX and contrast volume for predicting CIN.

Parameter	AUC	*P*	Cutoff	Sensitivity	Specificity
EASIX	0.908	<.001*	1.904	90.3%	86.2%
Contrast volume	0.833	<.001*	147.50 mL	74.2%	87.7%

AUC = area under the ROC curve, CIN = contrast-induced nephropathy, EASIX = Endothelial Activation and Stress Index, ROC = receiver operating characteristic.

* Significant at the.05 level according to ROC analysis.

**Figure 1.**
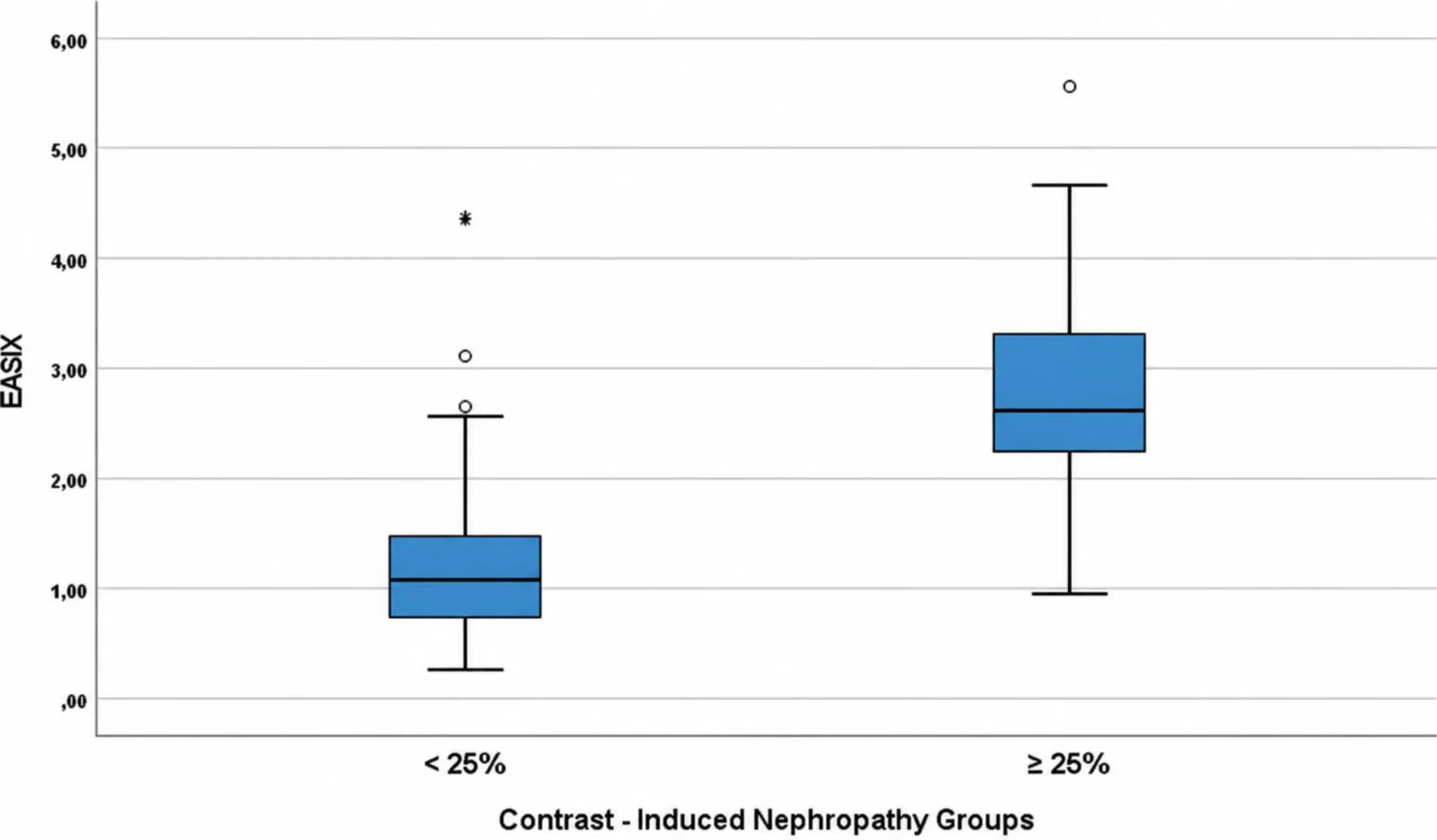
EASIX scores according to CIN groups. EASIX scores were compared between patients with <25% increase in serum creatinine (CIN negative) and those with ≥25% increase (CIN positive). The box plots illustrate the median (line within the box), interquartile range (box), and range (whiskers). Circles and asterisks denote outliers. CIN = contrast-induced nephropathy, EASIX = Endothelial Activation and Stress Index.

**Figure 2.**
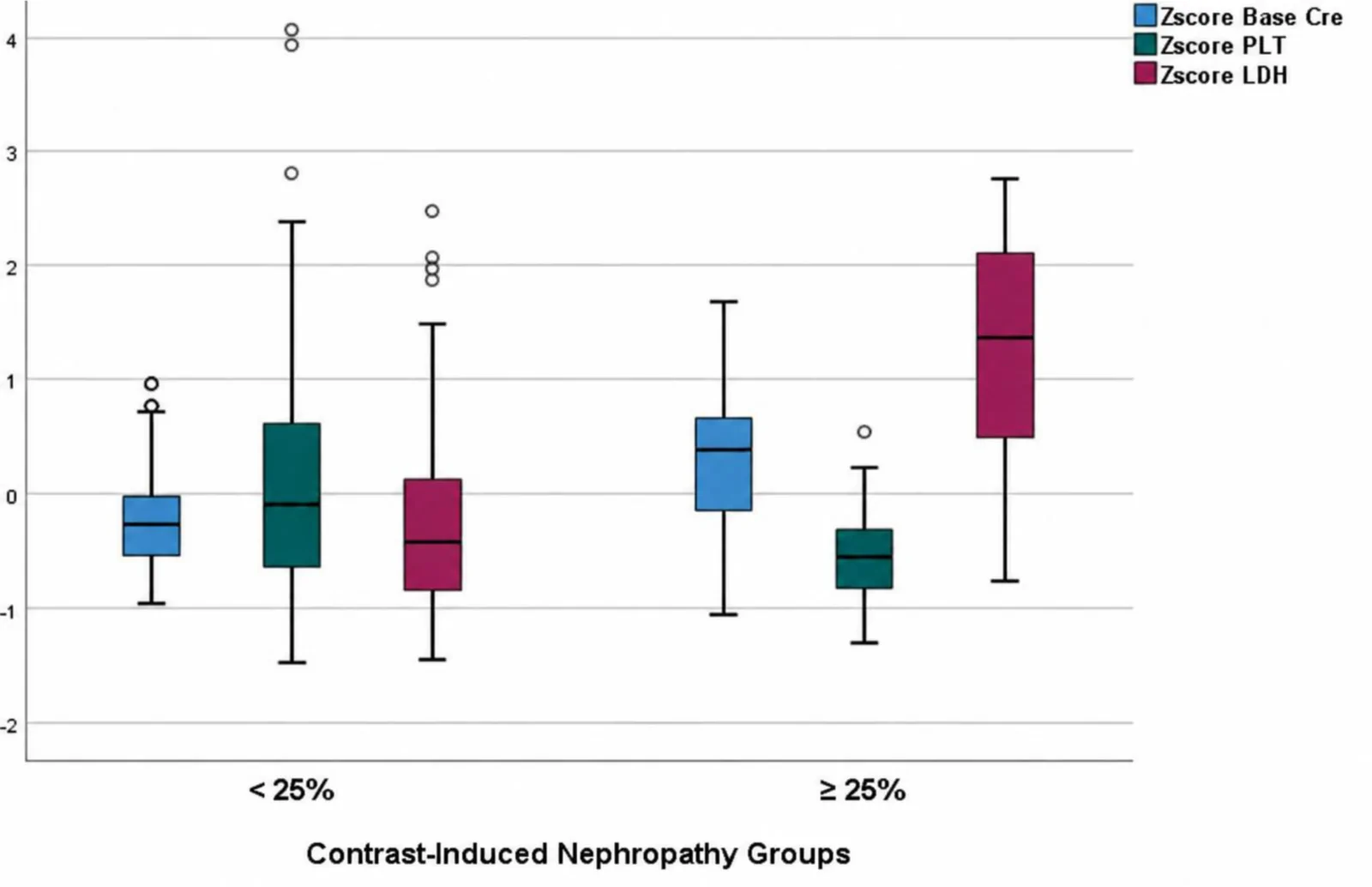
Standardized baseline creatinine, platelet count, and LDH values according to CIN groups. Box plots represent *z*-scores for baseline creatinine (blue), platelet count (green), and lactate dehydrogenase (purple) among patients with <25% increase in serum creatinine (CIN negative) versus ≥25% increase (CIN positive). Each box denotes the interquartile range, horizontal lines within boxes indicate the median, and whiskers depict the range. Circles indicate outliers. CIN = contrast-induced nephropathy, LDH = lactate dehydrogenase, PLT = platelets.

**Figure 3.**
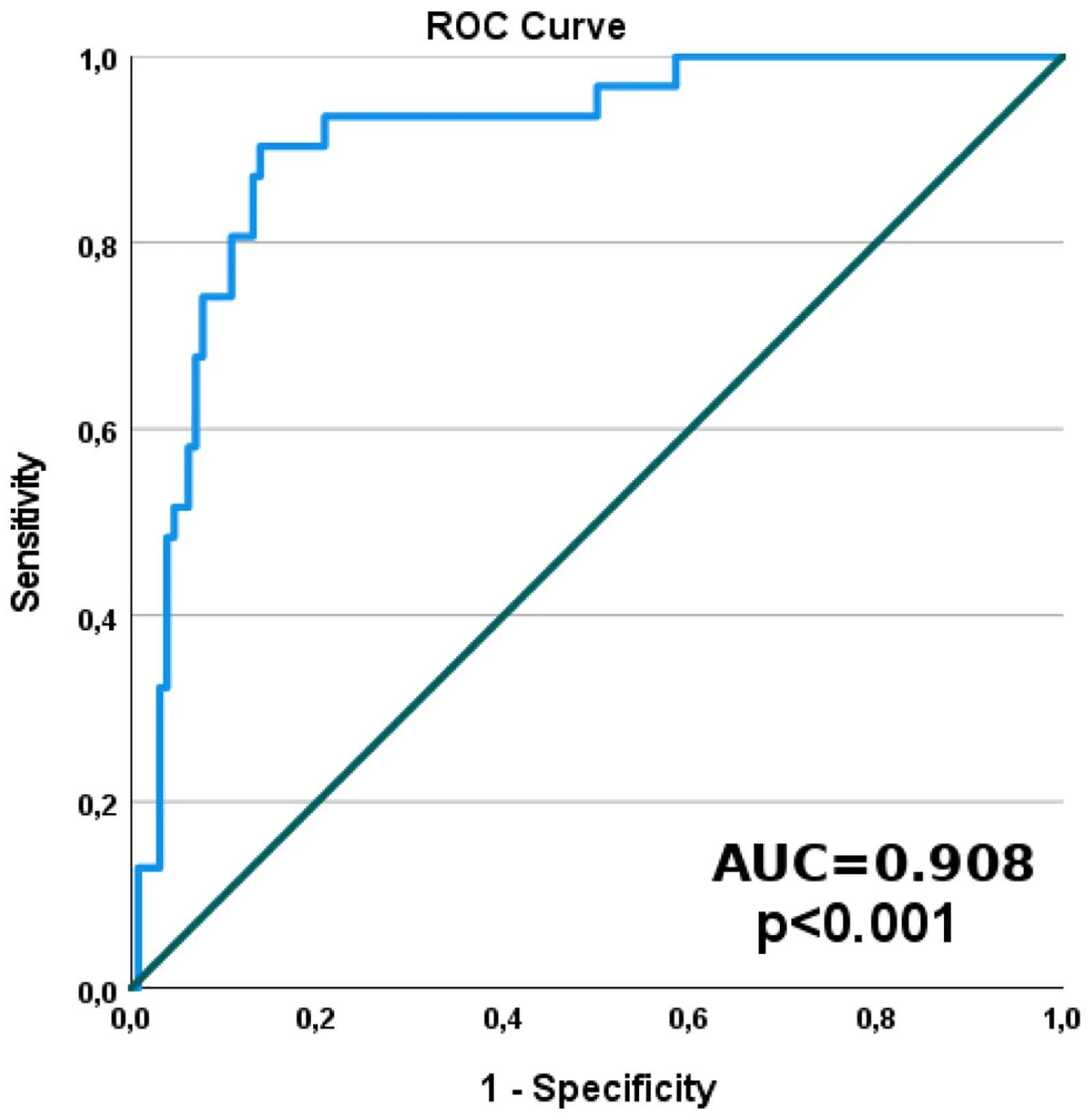
ROC curve of the EASIX score for predicting CIN. The receiver operating characteristic (ROC) analysis demonstrates the ability of the EASIX score to discriminate between patients who develop contrast-induced nephropathy (CIN+) and those who do not. The area under the curve (AUC) is 0.908, with *P* < .001, indicating excellent diagnostic performance. AUC = area under the curve, CIN = contrast-induced nephropathy, EASIX = Endothelial Activation and Stress Index, ROC = receiver operating characteristic.
